# Adolescent loneliness in the United States: Prevalence, sociodemographic correlates, and psychological health over time

**DOI:** 10.1016/j.ssmph.2026.101919

**Published:** 2026-04-02

**Authors:** Christopher D. Otmar, Jason M. Nagata

**Affiliations:** Department of Pediatrics, University of California, San Francisco, San Francisco, CA, USA

**Keywords:** Loneliness, Adolescence, Depression, Mental health, Sexual minority, Longitudinal

## Abstract

Loneliness is a public health concern, but its epidemiology in U.S. adolescence remains poorly described. Using data from the Adolescent Brain Cognitive Development (ABCD) Study (analytic N = 6067), we estimated the prevalence of loneliness at approximately age 14 and tested whether sociodemographic characteristics and psychological health were associated with later loneliness. Adolescents self-reported how often they felt lonely (“not lonely,” “sometimes,” “often”). Caregivers reported adolescents’ psychological symptoms (depression, anxiety, attention-deficit/hyperactivity, conduct, oppositional defiant, somatic) annually from baseline through Year 3. We estimated linear mixed-effects growth models to obtain person-specific intercepts (midpoint symptom level) and slopes (rate of change) and then used these trajectories as predictors of loneliness in multilevel ordinal models. Prevalence estimates indicated that 8.9% of adolescents reported being “often lonely,” 31.6% “sometimes lonely,” and 59.5% “not lonely.” In the fully adjusted multilevel model, females had higher odds of loneliness than males (OR = 1.88), and sexual-minority adolescents had higher odds than non-sexual-minority adolescents (OR = 2.81). Higher depressive symptom levels across late childhood and early adolescence were associated with greater odds of loneliness (OR = 1.29), and modest increases in conduct problems were also related to higher loneliness (OR = 1.12). The remaining symptom trajectories showed small or null associations after adjustment. The results from the present study suggest that, despite growing concern about an “epidemic” of loneliness, the experience is unevenly distributed by mid-adolescence, with elevated risk among female adolescents, sexual-minority adolescents, and those with heightened depressive symptoms.

## Highlights

• About 40% of U.S. adolescents reported at least some loneliness by approximately age 14.
• Female, sexual-minority, and American Indian/Alaska Native adolescents had higher odds of loneliness than their peers.
• Higher typical depressive symptom levels, as observed by caregivers, from late childhood to mid-adolescence were consistently associated with greater loneliness.

## 1 Adolescent loneliness in the United States

### 1.1 Prevalence, sociodemographic correlates, and psychological health over time

Loneliness is increasingly recognized as a public health concern that has consequences for both mental and physical well-being (U.S. Surgeon General’s Office, 2023). Among adults, chronic loneliness predicts depression, anxiety, poor sleep, and cardiometabolic disease (Cacioppo et al., 2011; Holt-Lunstad et al., 2015). Yet the etiology of these associations likely emerges far earlier (Qualter et al., 2015). Adolescence is a developmental period when self-evaluations become closely tied to peer belonging and feelings of acceptance (Dahl, 2004). Loneliness reflects perceived inadequacies in social connection, which may arise even when opportunities for participation are present (The Lancet Public Health, 2025). Further, loneliness may serve as one mechanism linking social inequality and chronic stress to later health risk, in part through sustained physiological dysregulation (Doyle & Link, 2024; Lau et al., 2025; The Lancet Public Health, 2025). Understanding who becomes lonely during this period, and why, is therefore central to prevention (von Soest et al., 2020).

Despite growing attention to loneliness as a health construct, most empirical work has emphasized adulthood or late life (Sheftel et al., 2024). This leaves the population-level epidemiology of adolescent loneliness in the United States underspecified. Yet, recent evidence indicates that global adolescent loneliness is not a rare condition but a widespread, and possibly stratified, experience. For example, in a pooled sample of more than 70 countries, it was estimated that roughly 12% of adolescents report frequent loneliness, with higher rates among females and adolescents in high-income countries (Hosozawa et al., 2025). These findings, together with evidence of increasing prevalence over the last decade (Twenge et al., 2021), suggest that loneliness might be tied to social experiences and identity. Hosozawa and colleagues (2025) called for large-scale, longitudinal, and cross-sectoral research to situate loneliness within broader determinants of health by integrating structural, psychological, and behavioral perspectives.

**Fig. 1:**
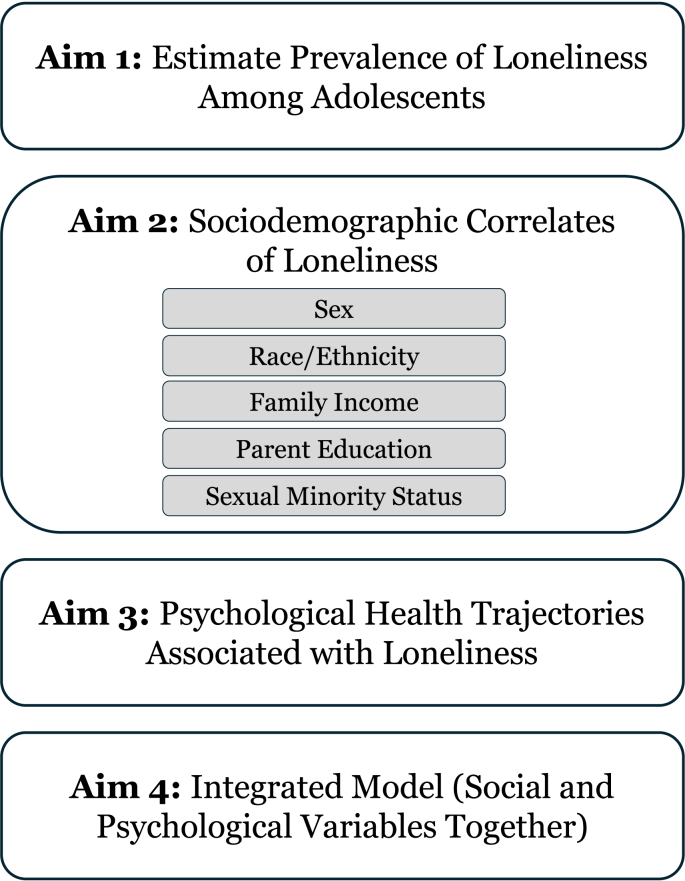
Study aims for estimating prevalence and predictors of loneliness in mid-adolescence.

The present study responds to that call by analyzing nationally weighted longitudinal data from the Adolescent Brain Cognitive Development (ABCD) Study to examine predictors of loneliness by mid-adolescence. Guided by a social determinants perspective (Adler et al., 1994), we examine whether loneliness is associated with sociodemographic characteristics (i.e., sex, race and ethnicity, parental education, family income) and identity (i.e., sexual minority status), consistent with disparities rooted in structural advantage and marginalization. Drawing on models of developmental cascades (Masten & Cicchetti, 2010), we also test whether trajectories of psychological health (i.e., depression, anxiety, attention-deficit/hyperactivity, conduct, oppositional defiant, and somatic) measured across four years exert spreading effects on adolescents’ social functioning and culminate in heightened loneliness by mid-adolescence. In doing so, this study examines whether adolescent loneliness reflects structural inequality, cumulative developmental spillover, or their intersection (see Fig. 1).

### 1.2 Sociodemographic correlates of adolescent loneliness

Loneliness reflects the perceived gap between desired and actual social connection (Hawkley & Cacioppo, 2010; Perlman & Peplau, 1981). Although defined as a psychological state, its prevalence varies systematically across demographic and socioeconomic groups (Barreto et al., 2024; Bower et al., 2023; Mullins et al., 1996). Accordingly, adolescent loneliness can be conceptualized not only as an individual experience, but as an outcome shaped by unequal structural conditions, in line with a social determinants perspective (Adler et al., 1994). Social determinants such as household income, parental education, and sexual minority identity organize the material and social environments in which adolescents participate (Henry et al., 2014; Matthews et al., 2019). These *upstream factors* influence access to shared activities, exposure to social support, and the ability to safely express identity within peer contexts. For example, family and neighborhood resources are a factor in whether adolescents can participate in school-based extracurricular activities and community programs or have access to safe gathering spaces and supportive institutional settings, all of which are key settings that belonging and peer relationships are built (e.g., Hjalmarsson, 2023; Morrow, 1999; O'Donnell et al., 2024; Tuominen & Tikkanen, 2023). As Braveman and colleagues (2011) describe, population health follows a stepwise gradient, such that each increase in social and economic resources is associated with improved health and participation, while disadvantage accumulates in those with fewer resources. Loneliness likely reflects this same gradient logic. If this argument holds, then adolescents positioned with fewer social and economic resources should face systematically higher risks of disconnection.

Population data from the United Kingdom show that loneliness is already stratified in adolescence (Siva, 2020). Eleven percent of children aged 10 to 15 years and fourteen percent of those aged 10 to 12 report being “often lonely,” and prevalence rises to forty percent among those aged 16 to 24 compared with twenty-seven percent of adults over 75 (Office for National Statistics, 2018a, 2018b). Economic resources appear particularly influential. Twenty-seven percent of children receiving free school meals report loneliness, and a U-shaped pattern shows that both low- and high-income adolescents are more likely to experience loneliness than those from middle-income households (Siva, 2020). These patterns indicate that loneliness associates with material conditions. Material scarcity limits participation in shared activities and reduces opportunities for everyday inclusion, while extreme affluence may introduce pressures related to competition, exclusivity, or social comparison (Siva, 2020). Although the mechanisms differ at opposite ends of the income distribution, the common thread is constrained relational integration.

Other sociodemographic characteristics may also shape feelings of loneliness. Racial and ethnic minority adolescents often encounter bias or discrimination that constrains social acceptance (Madsen et al., 2016). Sexual minority adolescents face stigma and a lack of affirming environments (Goldbach & Gibbs, 2017), which can restrict authentic disclosure and feelings of belonging (Alley et al., 2025; Diamond & Alley, 2022). Because stigma and concealment concerns can make everyday peer settings less predictable or safe, sexual minority adolescents may face additional barriers to routine inclusion. Parental education and income also provide intergenerational access to social capital (Morrow, 1999; Tuominen & Tikkanen, 2023), which could shape both academic participation and the perceived fit within peer networks. Sex differences may also contribute, such that female adolescents typically report loneliness more often than males (Siva, 2020), which could reflect socialized differences in emotional expression and expectations for relational connectedness.

By mid-adolescence, these multiple and intersecting social factors may have already structured who feels included. This framing positions adolescent loneliness within the same structural systems that govern disparities in physical and mental health by linking patterns of disconnection to broader systems of privilege and marginalization (Braveman & Gottlieb, 2014; Marmot & Wilkinson, 2005).

### 1.3 Longitudinal associations between psychological health and loneliness

A complementary body of work positions loneliness as a developmental process unfolding across multiple timescales (Lerner, 1987). Trajectories of stability, increase, and decline characterize the transition from childhood through adolescence (Qualter et al., 2013). Indeed, Laursen and Hartl (2013) describe adolescence as a particularly sensitive period. For instance, shifting companion networks and heightened self-focus magnify perceptions of social evaluation and ostracism. Comparable evidence exists in adulthood. Di Gessa and colleagues (2025) identified longitudinal patterns of loneliness (e.g., stable, increasing, and decreasing) that closely follow changes in health and social relationships. Deteriorating health predicted movement into lonelier trajectories, while improving health and supportive ties promoted recovery. In line with developmental cascade work (Masten & Cicchetti, 2010), these studies imply that problems emerging in one period of development can be carried forward and expressed in later-arising social tasks when contextual demands increase. The same pattern likely applies earlier in life, such that worsening mental health may initiate social withdrawal and disconnection (Rubin & Chronis-Tuscano, 2021), whereas resilience or symptom remission may stabilize social engagement (Fergus & Zimmerman, 2005).

Most research frames loneliness as a predictor of later psychological problems. For example, a recent study noted that adolescents reporting greater loneliness were more likely to develop depressive symptoms over time (Kwon et al., 2025). Yet this temporal ordering may also operate in reverse, with depression, anxiety, conduct, or attention deficit/hyperactivity symptoms preceding and shaping later loneliness (Luo et al., 2025). The current study asks whether psychological health, specifically, the level and rate of change in common psychopathological symptoms, associate with who reports being lonely by mid-adolescence. These trajectories capture both enduring differences and within-person change (Hoffman & Stawski, 2009), allowing us to test whether adolescents whose symptoms worsen over time are also those most likely to later report loneliness. In doing so, the analysis extends prior work by treating loneliness not only as an antecedent but also as a downstream outcome of cumulative psychological processes (Hosozawa et al., 2022; Mahon et al., 2006).

### 1.4 Integrating social and psychological predictors of loneliness

Until recently, research has examined social and psychological explanations of loneliness separately. During adolescence, these processes likely converge as structural contexts and emotional development become more tightly linked. Therefore, loneliness is best understood as the joint product of social and psychological factors that together shape adolescents' experiences of connection and disconnection. The growing sociology of loneliness is a necessary complement to psychological work. Van deVelde (2026) describes how loneliness has become a cross-disciplinary object of study yet notes that sociological engagement has lagged behind, even though loneliness is patterned by exclusion, poverty, life transitions, and stigma in ways that are unmistakably social (Yang, 2019). Drawing on Wood's description of loneliness as “a deeply personal feeling, yet inherently social and relative” (Wood, 1986; as cited in Van deVelde, 2026, p. 5), this framework implies that any examination of loneliness must attend both to the conditions adolescents inhabit and to the psychological processes through which those conditions are interpreted, remembered, and anticipated over time.

**Fig. 2:**
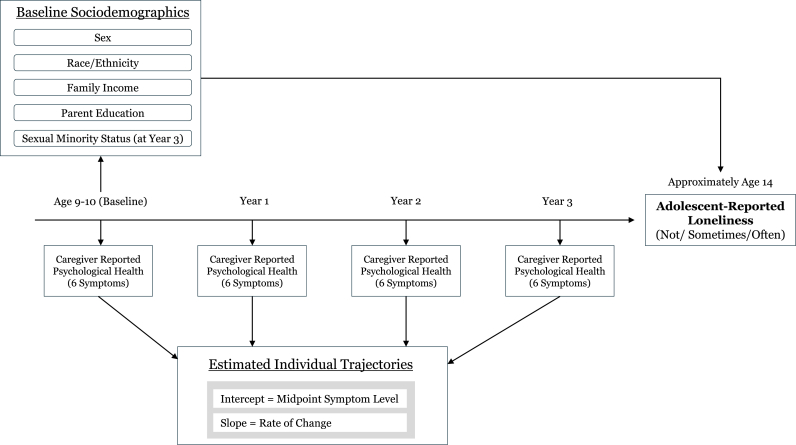
Conceptual model of adolescent loneliness using baseline sociodemographics and longitudinal psychological health trajectories **Note.** Caregiver-reported psychological health was assessed annually from baseline through Year 3 using CBCL DSM-oriented scales. Individual symptom trajectories were estimated with linear mixed-effects growth models with time coded 0–3 and centered at Year 1.5, such that intercepts represent estimated midpoint symptom levels and slopes represent annual rates of change. Person-specific intercept and slope empirical Bayes estimates for each symptom scale were carried forward as predictors of adolescent-reported loneliness at Year 4, modeled as an ordinal outcome.

The factors that produce loneliness are not purely social or purely psychological (Barjaková et al., 2023). Evidence shows that adolescents who experience loneliness often engage in behaviors and develop conditions that forecast poorer well-being across the life course. For example, Page (1990) found that lonely adolescents reported higher rates of substance use, restrictive dieting, and physical inactivity, which are patterns that prefigure adult health risk. Goosby and colleagues (2013) demonstrated that adolescent loneliness predicted later depression, poorer self-rated health, and elevated metabolic risk even after socioeconomic and family factors were considered. Matthews et al. (2024) further showed that adolescent loneliness forecasts poorer mental health, lower life satisfaction, and worse sleep and physical activity decades later. Viewing loneliness as the point where inequality and adaptation converge situates it within a developmental-epidemiological framework (Costello & Angold, 2015). The present analysis examines this intersection to clarify how enduring mental health patterns and social conditions jointly shape feelings of loneliness in mid-adolescence (see Fig. 2).

### 1.5 Study aims

The first aim is to estimate the prevalence of loneliness among adolescents in the United States. The second aim examines sociodemographic correlates of loneliness (i.e., sex, race and ethnicity, parental education, family income, and sexual minority status). The third aim assesses whether trajectories of psychological health over four years, capturing both level and rate of change in depression, anxiety, attention-deficit/hyperactivity, conduct, oppositional defiant, and somatic symptoms, are associated with loneliness by mid-adolescence. The final aim evaluates sociodemographic correlates and psychological health simultaneously to determine their independent and joint associations with loneliness.

## 2 Methods

Data were drawn from the Adolescent Brain Cognitive Development (ABCD) Study (Casey et al., 2018), a prospective cohort of U.S. children recruited at ages 9 to 10 from 21 research sites beginning in 2016. The ABCD Study follows participants annually and collects parallel caregiver and adolescent reports on health, behavior, and social context. The present analysis used deidentified data from baseline through the Year 4 assessment, which corresponds to mid-adolescence, approximately age 14. All analyses were conducted under an approved data use agreement, and all procedures followed ABCD guidelines for the use of baseline raked propensity weights.

### 2.1 Participants

Adolescents were eligible for inclusion if they had a self-reported loneliness score at Year 4 and at least two caregiver-reported Child Behavior Checklist (CBCL) assessments between baseline and Year 3. This criterion ensured that CBCL trajectories reflected change over time rather than single observations. After linking across waves, 6784 adolescents had valid Year 4 loneliness data. Because the final analysis combined baseline survey weights and inverse-probability-of-retention weights, the analytic sample for the models consisted of 6067 adolescents. Inverse-probability-of-retention weights were estimated in the baseline-eligible cohort (N = 11,522) and then applied to participants with observed Year 4 loneliness.

### 2.2 Measures

*Loneliness*. In Year 4, adolescents were asked how often they felt lonely, with responses coded 0 = “Not lonely,” 1 = “Sometimes,” and 2 = “Often.” This variable was analyzed as an ordered outcome. Single-item measures of loneliness are well established in large-scale and longitudinal studies for assessing perceived social disconnection (Ray, 2021; Ray & Shebib, 2022; van Roekel et al., 2018) and show strong correspondence with multi-item scales such as the UCLA Loneliness Scale (Russell, 1996).

*Sociodemographic variables*. Baseline covariates included caregiver-reported race and ethnicity (Asian, Black, Latino, American Indian/Alaska Native, Other/Multiracial, White), adolescents’ sex (caregiver-reported as female or male and coded 0 = male, 1 = female), household income (six categories, with ≥ $200,000 as the reference in regression models), and parental education (college degree or more vs. high school or some college). Sexual orientation was assessed at Year 3 and carried forward to the Year 4 loneliness assessment to preserve temporal ordering. Adolescents who identified as lesbian, gay, or bisexual were coded as sexual minority; all others were coded as non-sexual minority. In this coding scheme, odds ratios greater than 1 for sex indicate higher odds of loneliness for females relative to males.

*Psychological health*. Caregivers completed the Child Behavior Checklist (Achenbach & Ruffle, 2000) DSM-oriented scales at each wave from baseline through Year 3. Six scales were analyzed: attention deficit/hyperactivity, anxiety, conduct, depression, oppositional defiant, and somatic. Higher scores reflect more problems. To estimate multilevel growth, we treated the repeated CBCL assessments as Level-1 observations (time) nested within Level-2 units (adolescents), stacked all available CBCL data across waves, and coded time as assessment year (0-3), centered at the midpoint (Year 1.5) so that intercepts represented estimated symptom levels at the midpoint of baseline through Year 3.

*Psychological health trajectory estimation*. For each CBCL scale, we estimated a two-level linear growth model (multilevel linear mixed-effects model) treating time (centered assessment year) as Level 1 within adolescents at Level 2, with random intercepts and random slopes for time, using the ‘lme4’ (Bates et al., 2015) package with the bobyqa optimizer and increased iterations (Luke, 2017). From each model, we extracted empirical Bayes (EB) estimates (Casella, 1985) representing the person-specific intercept (estimated psychological health score at the midpoint of baseline through Year 3) and slope (rate of change from baseline through Year 3). This process yielded 12 parameters per adolescent (one intercept and slope for each of the six CBCL symptom categories). All six scales converged with random intercepts and random slopes. Because site was handled through the random intercept for site in the ordinal multilevel models rather than via site-residualization of the EB parameters, the same EB estimates were carried forward without additional site adjustment; for both the psychological health and integrated models, these EB parameters were standardized within the analysis sample to place all variables on a comparable scale.

This two-stage analytic approach, first estimating individual trajectories with mixed-effects models, then using those person-level EB estimates as predictors, was selected because a single joint model simultaneously incorporating six symptom processes, site effects, population weights, and an ordinal outcome was not readily estimable. Prior longitudinal work using EB estimates indicates that this method yields empirically derived person-specific estimates of stable symptom levels and rates of change (Cohen et al., 2005; Mohr et al., 2013), though these estimates are shrunken toward the sample mean and their uncertainty is not propagated to the next stage (Liu et al., 2021). Consequently, standard errors in the second-stage models may be somewhat underestimated.

### 2.3 Statistical analysis

Analyses proceeded in three phases. First, we examined weighted differences in loneliness across sociodemographic characteristics using Year 4 loneliness, site as the primary sampling unit, and the ABCD baseline raked propensity weights. We calculated weighted prevalence of each loneliness category and used Rao-Scott adjusted chi-square tests (Rao & Scott, 1987) to evaluate unadjusted differences by sex, race and ethnicity, income, parental education, and sexual minority status. We then estimated a two-level cumulative logit mixed-effects model predicting loneliness from these social and identity variables, with adolescents nested within sites (random intercept for site) and using the combined weight (baseline raked propensity weight multiplied by the inverse-probability-of-retention weight). Because Year 4 loneliness was an ordinal outcome and inclusion in the analytic sample depended on both the original ABCD sampling and subsequent retention, we treated these cumulative logit mixed models as model-based but incorporated combined design and retention weights, consistent with recommendations that survey-weighted multilevel and ordinal models can reduce bias when inclusion relates to the outcome or its predictors (Mitani et al., 2024; West et al., 2015).

Second, we examined associations between psychological health and loneliness in a multilevel logit model with a random intercept for site. In this psychological health model, Year 4 loneliness was regressed on the 12 standardized EB parameters to test whether adolescents with higher midpoint symptom levels or steeper increases over time were more likely to report loneliness at mid-adolescence, using the combined weight (baseline raked propensity weight multiplied by the inverse-probability-of-retention weight) and treating ABCD site as a level-2 clustering factor.

Finally, we estimated an integrated model that combined the social and psychological predictors while adjusting for attrition. To generate inverse-probability-of-retention weights, we modeled observation at Year 4 as a function of baseline sex, race and ethnicity, household income, parental education, sexual minority status, and site. Predicted probabilities were truncated between 0.01 and 0.99, inverted, and trimmed at the 1st and 99th percentiles; these inverse-probability weights were then multiplied by the ABCD baseline weight and normalized to have mean 1. Using this combined design, we fit a second multilevel logit model with a random intercept for site that included the 12 standardized CBCL EB parameters and all social and identity variables as fixed effects. All analyses were conducted in R version 4.4 using the 'lme4' (Bates et al., 2015), 'ordinal' (Christensen, 2018), and 'survey' (Lumley, 2004) packages. Reference groups were male sex, White race, highest income category (≥$200,000), college-educated parent, and non-sexual minority status.

## 3 Results

*Prevalence of loneliness.* Weighted estimates indicated that loneliness was common by mid-adolescence. Approximately 59.5% of adolescents reported being “not lonely,” 31.6% reported being “sometimes lonely,” and 8.9% reported being “often lonely” (Table 1). Thus, about 40% of adolescents reported at least some loneliness.

**Table 1:** Sample characteristics of U.S. Adolescents.

Characteristic	Unweighted n (%)	Weighted % (95% CI)∗
**Sex**		
Female	3025 (49.9)	49.7 (48.3–51.1)
Male	3042 (50.1)	50.3 (48.9–51.7)
**Race/Ethnicity**		
White (ref)	2970 (48.9)	49.5 (47.9–51.2)
Black	1035 (17.1)	15.8 (14.3–17.3)
Latino	1230 (20.3)	21.6 (20.0–23.2)
Asian	430 (7.1)	6.9 (5.8–8.0)
American Indian/Alaska Native	120 (2.0)	2.2 (1.6–2.8)
Other/Multiracial	282 (4.6)	4.0 (3.2–4.8)
**Household Income**		
< $25,000	680 (11.2)	10.5 (9.2–11.8)
$25,000 - < $50,000	910 (15.0)	15.7 (14.2–17.3)
$50,000 - < $75,000	1100 (18.1)	18.9 (17.2–20.6)
$75,000 - < $100,000	870 (14.3)	14.2 (12.8–15.6)
$100,000- < $200,000	1560 (25.7)	25.2 (23.5–26.9)
≥ $200,000 (ref)	947 (15.6)	15.5 (14.0–17.0)
**Parental Education**		
College degree or higher (ref)	4520 (74.6)	75.1 (73.4–76.8)
≤ High school/Some college	1547 (25.4)	24.9 (23.2–26.6)
**Sexual Minority Status**		
Non-Sexual Minority (ref)	5595 (92.2)	91.8 (90.5–93.1)
Sexual Minority	472 (7.8)	8.2 (6.9–9.5)
**Loneliness (Year 4)**		
Not Lonely	4054 (59.8)	59.5 (57.2–61.7)
Lonely Sometimes	2125 (31.4)	31.6 (29.7–33.5)
Lonely Often	605 (8.9)	8.9 (7.9–9.9)

**Note.** Analytic sample = 6067 adolescents with valid Year 4 loneliness and complete covariates. Weighted percentages and 95% CIs computed from ABCD baseline propensity weights with site-cluster adjustment. Loneliness was measured at Year 4. Percentages may not sum to 100 because of rounding.

**Fig. 3:**
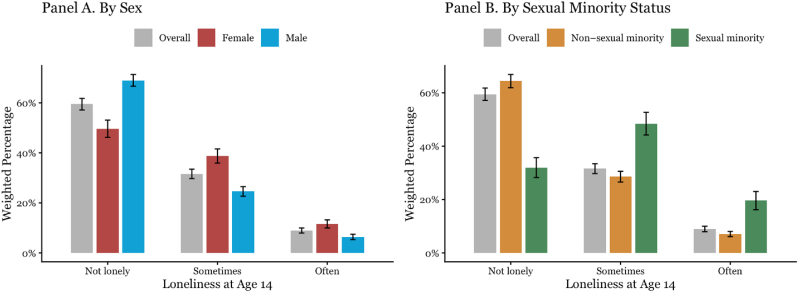
Prevalence of loneliness at approximately age 14 by sex and sexual minority status in study sample. **Note.** Bars show survey-weighted percentages; error bars show 95% confidence intervals. Loneliness was assessed at Year 4 of ABCD (m_age_ = 14 years) with a single self-report item (“How often do you feel lonely?“). “Overall” reflects the full weighted sample with non-missing loneliness and covariates. Sexual minority status (Panel B) was assessed in the wave immediately prior to the loneliness assessment; adolescents identifying as lesbian, gay, or bisexual were coded as sexual minority, all others as non-sexual minority. Estimates incorporate ABCD baseline weights and clustering by site.

*Sociodemographic correlates.* Weighted chi-square tests showed that loneliness differed significantly by sex, race and ethnicity, and sexual-minority status, but not by household income or parental education (Fig. 3). Females had higher odds of reporting more frequent loneliness than males (OR = 1.72, 95% CI = 1.54–1.96; see Table 2) and sexual-minority adolescents had nearly three times the odds of loneliness compared with non-sexual-minority adolescents (OR = 3.12, 95% CI = 2.66–3.67; Table 2). American Indian/Alaska Native adolescents also had elevated odds relative to White adolescents (OR = 1.51, 95% CI = 1.08–2.12). Differences for other racial and ethnic groups were smaller and not statistically reliable once sex and sexual-minority status were included. Neither household income nor parental education was associated with loneliness after adjustment.

**Table 2:** Weighted ordinal logistic regression of social determinants on loneliness at age 14 (ABCD study, N = 6067).

Predictor	OR	95 % CI	*t*	*p*
**Sex** (Female vs Male)	1.72	1.54 – 1.96	−8.90	<0.001
**Race/Ethnicity**				
Asian	1.11	0.86 – 1.44	0.78	0.436
Black	0.73	0.60 – 0.89	−3.11	0.002
Latino	1.04	0.86 – 1.26	0.37	0.712
American Indian/Alaska Native	1.51	1.08 – 2.12	2.38	0.017
Other	1	0.61 – 1.64	−0.02	0.986
**Household Income**				
< $25,000	1.04	0.79 – 1.36	0.27	0.789
$25,000 -<50,000	1.02	0.79 – 1.32	0.18	0.859
$50,000-<75,000	1.1	0.85 – 1.42	0.74	0.461
$75,000-<100,000	1.22	0.94 – 1.58	1.52	0.13
$100,000-<200,000	1.18	0.93 – 1.49	1.32	0.188
**Parental Education**	0.88	0.74 – 1.05	−1.34	0.182
**Sexual Minority Status**	3.12	2.66 – 3.67	13.96	<0.001

**Note.** Model thresholds: Not | Sometimes = 0.38 (SE = 0.12); Sometimes | Often = 2.39 (SE = 0.13). Reference categories were male, White, households with annual income ≥ $200,000, parents with a college degree or higher, and non-sexual-minority adolescents. Odds ratios greater than 1 indicate higher odds of reporting a more severe loneliness category. Estimates are derived from a two-level cumulative logit mixed-effects model with adolescents nested within sites and a random intercept for site. Models incorporate combined baseline propensity weights and inverse-probability-of-retention weights (trimmed and normalized).

*Psychological health model.* In the model including all 12 standardized CBCL parameters (Table 3), depressive problems over time were the most consistent correlates of loneliness. Adolescents with higher depressive intercepts, reflecting higher estimated depressive symptom levels at the midpoint of the baseline-Year 3 window, had greater odds of loneliness at Year 4 (OR = 1.30, 95% CI = 1.19–1.42) per 1-SD increase in the intercept parameter. Steeper increases in depressive symptoms across baseline through Year 3 were also associated with greater odds of loneliness (OR = 1.08, 95% CI = 1.00–1.15). In addition, steeper increases in anxiety (OR = 1.08, 95% CI = 1.01–1.15) and conduct problems (OR = 1.10, 95% CI = 1.01–1.19) were associated with higher odds of loneliness. By contrast, higher typical oppositional-defiant problems showed a small inverse association with loneliness (OR = 0.90, 95% CI = 0.82–1.00), and steeper increases in somatic symptoms were associated with lower odds of loneliness (OR = 0.91, 95% CI = 0.85–0.97), conditional on the full psychological health model. Intercepts and slopes for attention-deficit/hyperactivity and the remaining symptom parameters were not statistically significant.

**Table 3:** Associations between early-adolescent psychological health and loneliness at age 14 (N = 6067).

Predictor	β	SE	OR	95 % CI	*p*
**Attention-Deficit/Hyperactivity**					
Intercept	−0.01	0.04	0.99	0.92–1.07	0.853
Slope	0.03	0.03	1.04	0.97–1.11	0.294
**Anxiety**					
Intercept	0.01	0.04	1.01	0.93–1.09	0.838
Slope	0.07	0.03	1.08	1.01–1.15	0.021
**Conduct**					
Intercept	0.07	0.05	1.07	0.97–1.18	0.181
Slope	0.09	0.04	1.1	1.02–1.19	0.02
**Depression**					
Intercept	0.26	0.04	1.3	1.19–1.42	<0.001
Slope	0.07	0.03	1.08	1.01–1.16	0.036
**Oppositional Defiant**					
Intercept	−0.1	0.05	0.9	0.82–1.00	0.045
Slope	−0.01	0.04	0.99	0.92–1.07	0.692
**Somatic**					
Intercept	−0.04	0.04	0.96	0.89–1.03	0.201
Slope	−0.1	0.03	0.91	0.85–0.97	0.002

**Note.** Standardized logit coefficients (β) and odds ratios (OR) from a two-level cumulative logit mixed-effects model predicting Year 4 loneliness (Not, Sometimes, Often) from person-specific intercepts (mid-window symptom level across baseline–Year 3) and slopes (annual change) of psychological health scales. Adolescents were nested within sites with a random site intercept. Models incorporate combined baseline propensity weights and inverse-probability-of-retention weights (trimmed and normalized). Positive coefficients indicate higher odds of reporting a more severe loneliness category. p values are two-tailed.

*Integrated model.* When population and retention weights and all social and psychological predictors were included simultaneously, the sociodemographic factors remained consistent with the prior models (Table 4). Females had higher odds of loneliness than males (OR = 1.88, 95% CI = 1.66–2.13), and sexual-minority adolescents continued to show higher odds (OR = 2.81, 95% CI = 2.39–3.30). American Indian/Alaska Native adolescents had higher odds of loneliness than White adolescents (OR = 1.48, 95% CI = 1.05–2.0). Most psychological variable effects were attenuated once survey design and attrition were accounted for. The depressive intercept remained significant and positive (OR = 1.29, 95% CI = 1.18–1.41), meaning that adolescents with higher typical depressive symptom levels were more likely to report loneliness. Conduct-problem slopes also remained statistically associated with loneliness, but with a relatively small effect (OR = 1.12, 95% CI = 1.03–1.21). In contrast, depressive slopes and the remaining symptom parameters were not significant in the fully adjusted model.

**Table 4:** Integrated multilevel model predicting loneliness at age 14 (ABCD study, N = 6067).

Predictor	β	SE	OR	95% CI	z	p
**Sociodemographic Characteristics**						
Female (vs Male)	0.63	0.06	1.88	1.66–2.13	9.75	<0.001
Asian	0.16	0.13	1.17	0.90–1.52	1.20	0.23
Black	−0.27	0.10	0.77	0.63–0.94	−2.60	0.01
Latino	0.05	0.10	1.05	0.87–1.26	0.49	0.62
American Indian/Alaska Native	0.39	0.17	1.48	1.05–2.08	2.27	0.02
Other	0.02	0.25	1.02	0.62–1.68	0.08	0.93
< $25,000	−0.02	0.15	0.98	0.74–1.30	−0.12	0.90
$25,000–<50,000	−0.05	0.13	0.95	0.73–1.23	−0.35	0.73
$50,000–<75,000	0.06	0.13	1.07	0.83–1.38	0.48	0.63
$75,000–<100,000	0.16	0.13	1.17	0.90–1.53	1.18	0.24
$100,000–<200,000	0.16	0.12	1.17	0.92–1.48	1.29	0.20
Parent Education (HS or some college)	−0.10	0.09	0.90	0.75–1.08	−1.09	0.28
Sexual Minority	1.03	0.08	2.81	2.39–3.30	12.43	<0.001
**Psychological Health Trajectories**						
ADHD Intercept	−0.04	0.04	0.96	0.88–1.05	−1.06	0.29
ADHD Slope	0.00	0.03	1.00	0.94–1.07	−0.09	0.93
Anxiety Intercept	−0.01	0.04	0.99	0.91–1.08	−0.12	0.90
Anxiety Slope	0.03	0.03	1.03	0.97–1.10	1.00	0.32
Conduct Intercept	0.09	0.05	1.10	0.99–1.22	1.79	0.07
Conduct Slope	0.11	0.04	1.12	1.03–1.21	2.69	0.01
Depression Intercept	0.25	0.05	1.29	1.18–1.41	5.53	<0.001
Depression Slope	0.03	0.04	1.04	0.97–1.12	0.97	0.33
Oppositional Intercept	−0.05	0.05	0.95	0.86–1.05	−0.92	0.36
Oppositional Slope	0.02	0.04	1.02	0.95–1.10	0.40	0.69
Somatic Intercept	0.02	0.04	1.02	0.95–1.10	0.48	0.63
Somatic Slope	0.02	0.03	1.02	0.96–1.09	0.47	0.64

Note. Standardized logit coefficients (β) and odds ratios (OR) from a two-level cumulative logit mixed-effects model predicting Year 4 loneliness (Not, Sometimes, Often) from sociodemographic characteristics and person-specific intercepts (mid-window psychological health level across baseline–Year 3) and slopes (annual change) of CBCL DSM-oriented scales. Adolescents were nested within sites with a random site intercept. Models incorporate combined baseline propensity weights and inverse-probability-of-retention weights (trimmed and normalized). Model thresholds: Not | Sometimes = 0.31 (SE = 0.12); Sometimes | Often = 2.36 (SE = 0.13). Reference categories: male, White, household income ≥ $200,000, parent with college degree or higher, and non-sexual-minority adolescents. Odds ratios greater than 1 indicate higher odds of reporting a more severe loneliness category.

*Sensitivity analyses.* In sensitivity analyses, we first examined psychological health variables and EB reliability for the growth parameters. We observed that most participants contributed four waves of CBCL data (10,061 with 4 waves; 1461 with 2 to 3 waves) and that EB intercepts were generally highly reliable (median = 0.80–0.91 across scales), whereas slope reliabilities were more modest (median = 0.21–0.37). These diagnostics suggest that the person-specific intercepts can reasonably be treated as more stable, or trait-like, symptoms, whereas slopes are better viewed as approximations of change in psychological health rather than fine-grained individual trajectories. We then refit all three multilevel ordinal models in the subset of adolescents with complete four-wave trajectories (N = 5944; versus N = 6067 in the main models) and found that the pattern of associations was very similar: higher depressive intercepts remained associated with greater loneliness (ORs = 1.26–1.30 across psychological health-only and integrated models), and sexual-minority status continued to show a large association with loneliness (ORs = 2.8–3.1), with comparable sex and race effects. Overall, the wave-count, EB reliability, and four-wave restriction analyses support the robustness of the primary conclusions regarding chronic depressive symptoms and sexual-minority status as correlates of adolescent loneliness, while also indicating that inferences about symptom slopes should be interpreted with greater caution.

## 4 Discussion

In this longitudinal cohort in the United States, loneliness by mid-adolescence was common but not universal. Loneliness differed by sex, sexual-minority status, and race and ethnicity, and we did not find evidence of disparities by household income or parental education. When psychological health was incorporated, depressive symptoms showed the clearest association with loneliness. In the model focused on psychological health, both higher depressive symptom levels and steeper increases over late childhood were associated with greater loneliness. However, once sociodemographic characteristics were included and the analyses incorporated population and retention weights, only depressive symptom levels remained significant, whereas depressive symptom change over time did not.

### 4.1 A note on the epidemic of loneliness

Approximately sixty percent of adolescents reported “not lonely,” one-third described feeling “sometimes lonely,” and fewer than one in ten reported feeling “often lonely.” This distribution matches recent cross-national work in which a minority report frequent loneliness and a larger group reports intermittent or situational loneliness (Hosozawa et al., 2022). In practical terms, a sizable segment of adolescents is already describing an unmet need for connection by mid-adolescence. Mid-adolescence represents a period in life that should, in principle, be socially dense (Hartup & Stevens, 1999). In that sense, these estimates are aligned with broader claims in social neuroscience and public health that loneliness is a common experience rather than a rare condition, thereby extending more adult-focused summaries to an earlier developmental window (Cacioppo et al., 2011; Office of the Surgeon General, 2023).

However, characterizing adolescent loneliness as an “epidemic” captures public concern but risks obscuring how loneliness is actually distributed and when in the life course it frustrates well-being. In this adolescent cohort, most youth reported little or no loneliness, a sizable minority experienced it intermittently, and a smaller subgroup reported frequent or persistent loneliness. This pattern is consistent with Hall et al.'s (2025) recent evidence in an adult sample that social ill-being is elevated in emerging adulthood but does not engulf entire age groups, and that loneliness can co-occur with high levels of connection, companionship, and support. In our younger sample, loneliness is already concentrated among adolescents with sustained depressive symptoms and those who might anticipate exclusion or feel socially different (e.g., sexual-minority youth), before the major role transitions and ontological insecurity that Hall et al. argue characterize emerging adulthood. Put differently, the stratified “ambivalent” social health pattern Hall et al. describe in young adults appears to have roots in earlier adolescence, where unequal access to belonging and ongoing psychological challenges are already sorting adolescents into groups with differing likelihoods of feeling lonely.

### 4.2 Sociodemographic correlates

In this cohort, female adolescents reported greater loneliness. This pattern aligns with evidence that girls are, on average, more likely than boys to report frequent loneliness, although the size (and in some cases the direction) of this gap shifts across countries (Igami et al., 2023). Trend data from high-income school-based samples indicate that school loneliness has increased for both girls and boys over the last decade, with a sharper rise and a larger shift into high-loneliness categories among girls (Twenge et al., 2021). Experience sampling work with early adolescent girls suggests that their days are already dense with peer contact, but the felt quality of those interactions fluctuates sharply, and low-connection moments are precisely when negative peer encounters remain most salient (Do et al., 2025). Longitudinal analyses following adolescents into young adulthood indicate that when girls do feel lonely, the consequences extend beyond mood and into physical health; for example, adolescent loneliness predicts poorer self-rated health and higher risk of overweight and obesity in young adult women (Goosby et al., 2013). At the same time, Singh's (2021) mapping of girlhood scholarship reminds us that claims about “girls” are always grounded in particular methodological and social locations, and that which girls are centered (e.g., race, class, sexuality, and geography) shapes what becomes visible. Accordingly, our finding that female adolescents report greater loneliness by age fourteen is best understood not as an essential feature of girlhood, but as one structured outcome of how contemporary U.S. schools, peer cultures, and identity hierarchies organize girls' opportunities for connection and belonging.

Sexual minority adolescents also reported greater loneliness. This early separation likely reflects the cumulative weight of social learning that begins before identity disclosure (e.g., subtle exclusion, heteronormative cues, and perceived difference) that signal nonbelonging (Hart et al., 2018; Savin-Williams & Diamond, 2000). Because our measure captures adolescents who already identify as gay, lesbian, or bisexual by early adolescence, these youth may represent a subgroup with earlier awareness or disclosure, whereas others who are questioning or not yet out are classified as non-sexual minority in our data, likely yielding conservative estimates of disparity. Longitudinal studies show that such experiences, recalled later as early social exclusion, are linked in young adulthood to higher minority stress, stronger self-blame and disengagement, and greater social anxiety (Otmar & Merolla, 2024). Qualitative research further indicates that LGBTQ individuals describe hypervigilant monitoring of others and concealment of aspects of the self as ongoing strategies to manage anticipated rejection (Rostosky et al., 2022). Over time, these adaptive but costly strategies can make ordinary social interaction feel effortful and depleting (Hall et al., 2023), providing one plausible route through which sexual-minority adolescents come to experience more loneliness, particularly when early exclusion and minority stress accumulate.

Finally, American Indian/Alaska Native adolescents had higher odds of loneliness than White adolescents. There is limited empirical work on loneliness in Indigenous communities, but emerging studies in the Blackfeet Nation show that loneliness is related to poorer sleep and worse mental health and is inversely associated with spirituality and cultural connection. (Henderson-Matthews et al., 2025; John-Henderson et al., 2022). The pattern evidenced in this study is consistent with scholarship on structural and historical marginalization in Indigenous communities, in which colonization, historical loss, ongoing discrimination, and geographic and social isolation constrain opportunities for stable connection (Barry et al., 2025; Dickerson et al., 2024; Henderson-Matthews et al., 2025). Although the present data cannot identify specific mechanisms, elevated loneliness among American Indian/Alaska Native adolescents is plausibly linked to structural conditions and points to the need for tribally led work that can define and address loneliness using Indigenous frameworks for relatedness and well-being.

### 4.3 Psychological health over time

In models that focused on psychological health, higher levels of depressive problems and steeper increases in depressive, anxiety, and conduct problems from late childhood into early adolescence were associated with greater loneliness, whereas increasing somatic complaints were associated with lower loneliness. Yet, in the integrated model, these slope associations largely diminished, leaving a consistent association for chronic depressive problems and a smaller association for increasing conduct problems. In other words, stable elevations in depressive symptoms, more than changes over time in broader psychological health, were most consistently linked to who reported loneliness by mid-adolescence.

Much of adolescent research has conceptualized loneliness as a precursor to later depression, with lonely adolescents at greater risk for subsequent depressive symptoms and related problems (Dunn & Sicouri, 2022; Schwartz-Mette et al., 2020). Here, the analyses were organized in the reverse temporal ordering, using caregiver-reported psychological health from late childhood to predict adolescents’ own reports of loneliness around age fourteen. This structure does not establish causation, but it does indicate that persistent depressive problems, as observed by caregivers, are already in place before adolescents describe themselves as lonely. A reasonable interpretation is that sustained depressive symptoms alter social behavior and perception, which may then foster withdrawal (Qualter et al., 2015), negative construal of ambiguous cues (Lin et al., 2019; Prieto-Fidalgo et al., 2023), and difficulty maintaining relationships (Merolla et al., 2023), which may then be experienced as loneliness. Loneliness, in turn, may coincide with renewed depressive affect. Future longitudinal studies should formally model these reciprocal pathways in adolescence (Kristensen et al., 2023).

### 4.4 Limitations

Limitations are also important to specify. It is important to note that this research hinges on a single ordered item capturing a general sense of disconnection. Although consistent with surveillance practice, it is unable to differentiate transient from chronic experiences (van Roekel et al., 2018) and potentially sensitive to current mood. Although this measure revealed systematic variation and was sufficient for design-based modeling, it aggregates distinct experiences (e.g., social versus emotional loneliness, peer-versus family-based isolation, and transient versus persistent disconnection) into a single response (Qualter & Munn, 2002; Russell et al., 1984; Walsh et al., 2025). These forms likely characterize different processes and vulnerabilities, including friendship dissolution (Leclerc Bedard et al., 2025) and structural exclusion (Büttner et al., 2025). A one-item measure cannot distinguish among these pathways or identify which social contexts are most consequential. Advancing this work will require multi-item, form-specific assessments in large population samples to test whether the observed differences by sex, sexual-minority status, and American Indian/Alaska Native identification converge within particular types of loneliness.

Psychological symptoms were reported by caregivers, whose awareness of internalizing problems varies and may attenuate some associations. The developmental window of ages nine to fourteen is narrow, so the findings do not extend to later contexts such as first romantic relationships the transition from high school to college, or early employment. Because the models are observational, unmeasured factors (e.g., school climate, peer victimization, disability) could shape both symptom histories and loneliness.

Analytically, the study modeled longitudinal symptom trajectories and incorporated design-based weighting to account for attrition and population structure. This approach improves generalizability but relies on a two-stage strategy in which empirical Bayes random effects from the growth models are treated as predictors in a second set of ordinal models. As Liu and colleagues (2021) note, EB estimates are shrunken toward the sample mean and their uncertainty is not propagated into follow-up models, so coefficients in such second-stage regressions are likely to be attenuated, particularly when EB reliability is modest. In the present study, most intercepts achieved high reliability, whereas slopes were less precise, which suggests that inferences involving slope parameters warrant greater caution. Because sexual orientation was first assessed at Year 3 and entered the retention model, anchoring the retention weights at that wave aligns with the timing of the Year 4 loneliness outcome but may introduce some selection if early attritors differed systematically by identity.

Consistent with work on multilevel and survey-weighted ordinal models, incorporating estimated inclusion probabilities is expected to reduce bias in cumulative logit and related multilevel estimates when sampling or retention depends on the outcome or its determinants (Mitani et al., 2024; West et al., 2015), yet the absence of separate site-level weights and the reliance on baseline covariates rather than direct conditioning on loneliness categories mean that some residual design-related bias cannot be ruled out. Finally, although the ABCD Study used an epidemiologically informed, school-based sampling strategy and baseline propensity weights to approximate U.S. demographics, the cohort should not be treated as fully representative of all U.S. adolescents for the purpose of estimating population prevalence (Compton et al., 2019). Our estimates are therefore best interpreted as characterizing patterns within this large, demographically diverse U.S. cohort rather than as definitive national or cross-national prevalence benchmarks.

We conclude with a consideration of practical applications and priorities for future work. We focus on three potential applications of the current findings. First, because loneliness was more closely linked to adolescents’ typical level of depressive symptoms than to modest variability in symptom change, routine screening for both depression and feelings of loneliness in pediatric and school settings should be treated as standard practice rather than reserved for periods of acute crisis, with clear referral pathways to evidence-based care. Second, the elevated odds of loneliness among sexual-minority adolescents point to the importance of school and community settings that are structurally affirming (e.g., well-supported GSAs, inclusive curricula, explicit protections for LGBTQ students), which can reduce everyday vigilance and social risk without placing the burden solely on individual coping. Third, higher loneliness among American Indian/Alaska Native adolescents—though estimated with less precision—suggests that loneliness should be understood in relation to ongoing colonial and structural conditions, with tribally led initiatives defining what social connection and support look like in context.

## CRediT authorship contribution statement

**Christopher D. Otmar:** Writing – review & editing, Writing – original draft, Visualization, Methodology, Formal analysis, Conceptualization. **Jason M. Nagata:** Writing – review & editing, Funding acquisition.

## Consent to participate

Parental consent and youth assent were obtained in accordance with ABCD procedures.

## Consent to publish

Not applicable.

## Role of funder sponsor

The funders had no role in the study analysis, decision to publish the study, or the preparation of the manuscript.

## Study registration

This is a secondary observational analysis without an intervention trial.

## Analytic plan registration

Not preregistered.

## Materials availability

Not applicable. No novel instruments or lab materials beyond standard questionnaires.

## Reporting standards and required descriptions

We followed all STROBE checklist guidelines for observational cohort studies and state adherence in the Methods.

## Funding/support

The research was supported by the National Institutes of Health (K08HL159350, R01MH135492, and R01DA064134). The ABCD Study was supported by the National Institutes of Health and additional federal partners under award numbers U01DA041022, U01DA041025, U01DA041028, U01DA041048, U01DA041089, U01DA041093, U01DA041106, U01DA041117, U01DA041120, U01DA041134, U01DA041148, U01DA041156, U01DA041174, U24DA041123, and U24DA041147. A full list of supporters is available at https://abcdstudy.org/about/federal-partners/. A listing of participating sites and a complete listing of the study investigators can be found at https://abcdstudy.org/principal-investigators.html. ABCD consortium investigators designed and implemented the study and/or provided data but did not necessarily participate in analysis or writing of this report.

## Declaration of competing interest

The authors declare that they have no known competing financial interests or personal relationships that could have appeared to influence the work reported in this paper.

## Data Availability

Data used in the preparation of this article were obtained from the ABCD Study (https://abcdstudy.org), held in the NIH Brain Development Cohorts (NBDC) Portal.
